# Supplementary material for: Prosit-XL: enhanced cross-linked peptide identification by fragment intensity prediction to study protein interactions and structures
Source: Nat Commun. 2025 Jul 1;16:5429. doi: 10.1038/s41467-025-61203-4 (PMC12214610; doi:10.1038/s41467-025-61203-4)
Supplement: Supplementary file 1 — Supplementary Information [file 41467_2025_61203_MOESM1_ESM.pdf]

Supplementary Figure 1

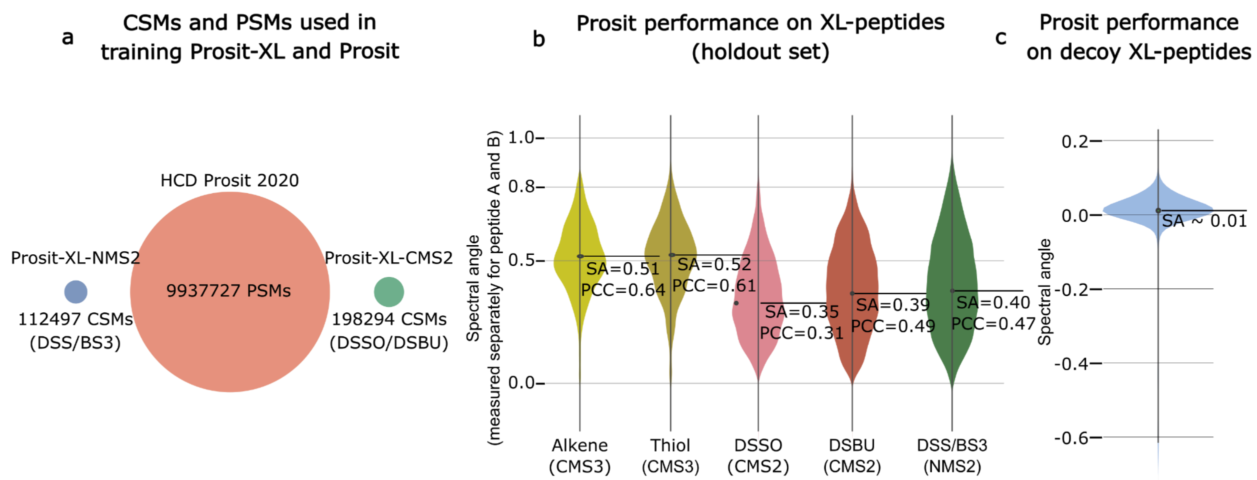

Supplementary Fig. 1 | Comparison of available training data of Prosit and Prosit-XL and performance evaluation of Prosit on XL-peptides. a) Comparison of the number of CSMs and PSMs used in training Prosit-XL and Prosit. **b)** Violin plot showing the prediction accuracy of Prosit model for CMS3, CMS2, and NMS2 on the holdout set across 5 different cross-linker types: CMS3-Alkene, CMS3-Thiol, CMS2-DSSO, CMS2-DSBU, and NMS2-DSS/BS3. The black solid line and corresponding numbers indicate the median spectral angle (SA) and Pearson correlation coefficient (PCC). The prediction performance was assessed separately for peptides A and B (PSM level). **c)** Violin plot showing the prediction accuracy of the Prosit model on decoy XL-peptides from the synthetic peptide dataset (DSSO) from replicate one, labeled as 20210203_QExHFX3_RSLC10_DSSO_mainlib_rep1.raw. The measurement was performed exclusively on peptide A.

Supplementary Figure 2

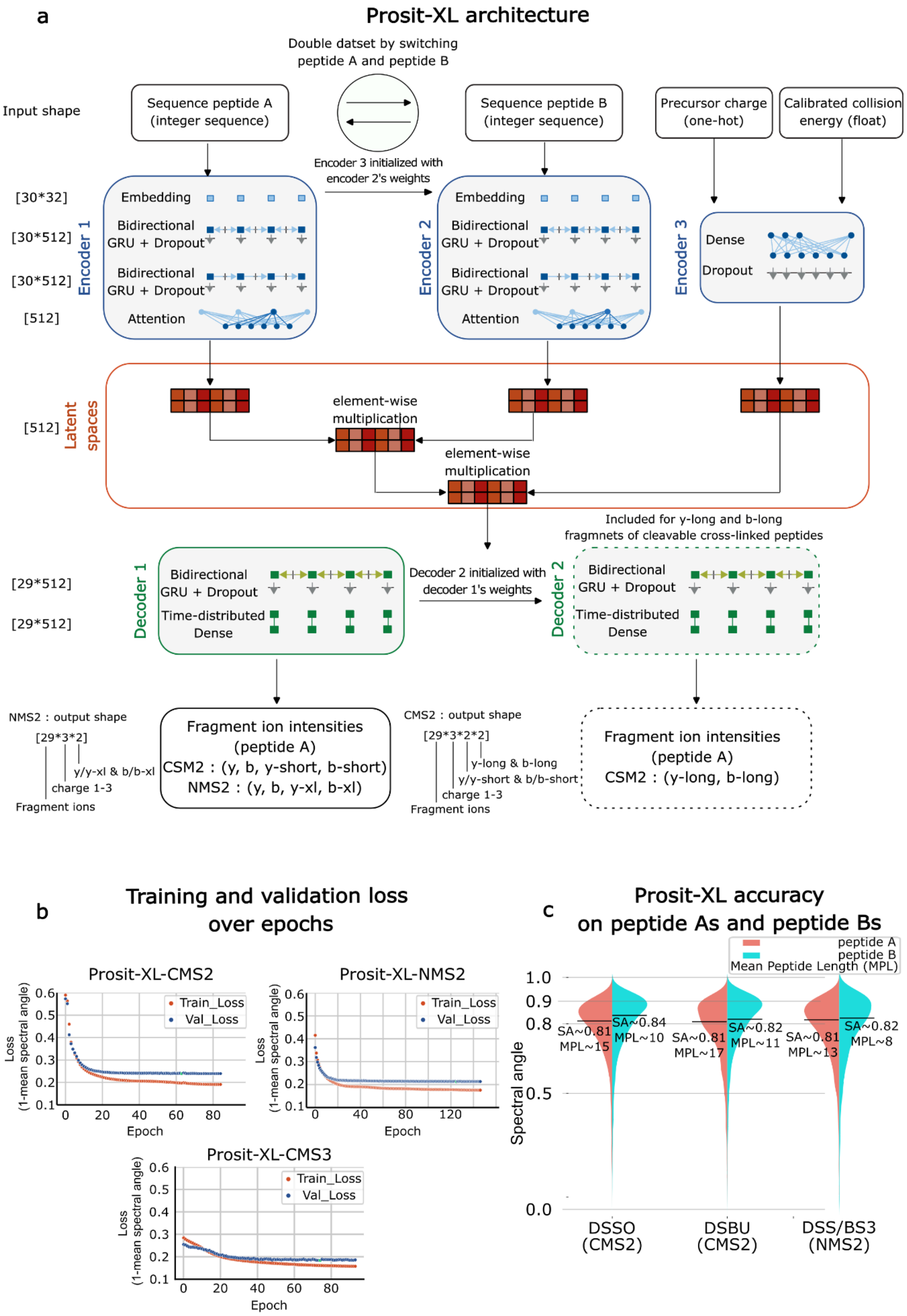

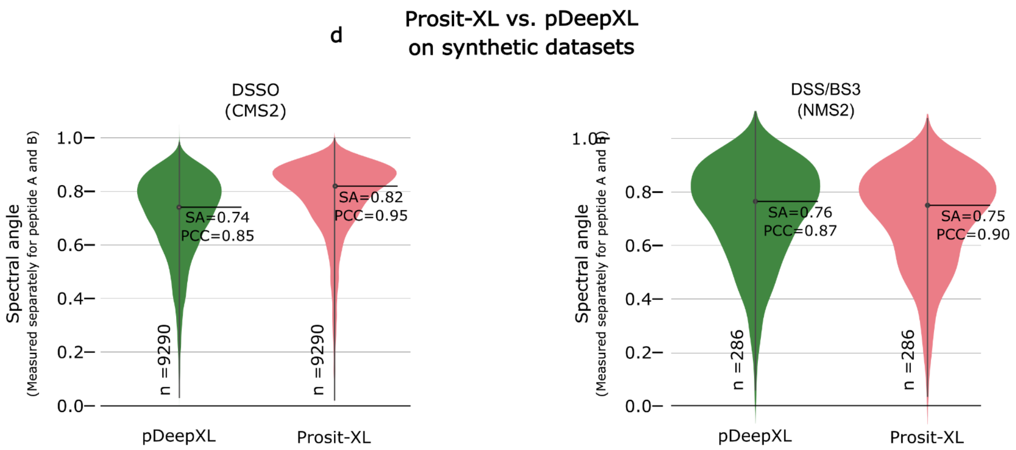

Supplementary Fig. 2 | Prosit-XL model architecture, performance and training characteristics. a). The model takes precursor charge, normalized collision energy and the peptide sequence A and B as input. The encoders for the peptide sequence A and B (encoder 1 and 2) are split in an embedding layer connected to 2 bi-directional recurrent neural networks (BDN) with gated recurrent memory (GRU) units and an attention layer. The encoder 3 consists of one dense layer for precursor charge and normalized collision energy. The encoder 1 and 2 representations are element-wise multiplied and then the resulting output and encoder 3 representations are element-wise multiplied for a fixed size latent space representation. The decoder 1 and 2 for fragment ion intensity prediction consists of one bidirectional GRU resulting in 6 predictions for up to 29 fragmentation positions. The decoder 2 is specifically designed to cover fragments containing the long part of the cleavable crosslinker as modification. **b)** Training and validation loss over epochs. The loss function is calculated by the mean of 1 minus the spectral angle between predicted and experimental fragment ion intensities The orange and blue points show training and validation loss, respectively. **c)** Violin plot comparing the prediction accuracy of Prosit-XL models on peptide As (red) and peptide Bs (light blue) for CMS2 and NMS2 on the holdout set across 3 different cross-linker types: CMS2-DSSO, CMS2-DSBU, and NMS2-DSS/BS3. The black solid line and corresponding numbers indicate the median spectral angle (SA) and the mean of peptide length (MPL). **d)** Violin plot comparing the prediction accuracy of Prosit-XL and pDeepXL models on external unseen synthetic datasets using DSSO and DSS/BS3 as cross-linkers. The number of underlying spectra (n) is indicated at the bottom. The black solid line and corresponding numbers indicate the median spectral angle and Pearson correlation.

Supplementary Figure 3

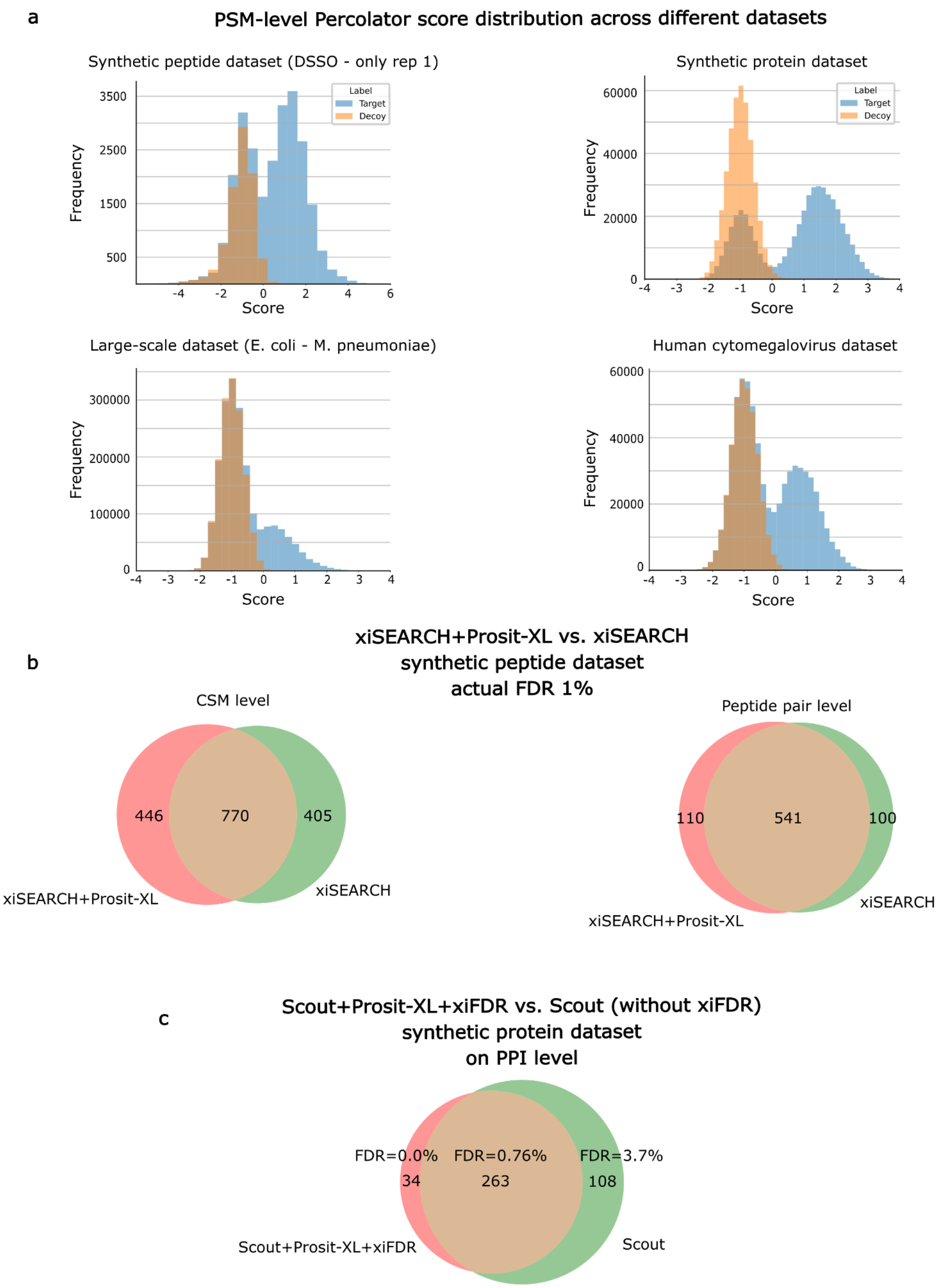

Supplementary Fig. 3 | Validation of data-driven rescoring approach. a) PSM-level Percolator score distribution across different datasets including synthetic peptide dataset (upper-left, only rep1), synthetic protein dataset (upper-right), large-scale dataset (*E. coli* - *M. pneumoniae*, bottom-left) and human cytomegalovirus dataset (bottom-right). Targets and decoys are represented by blue and orange, respectively. **b)** Comparison of xiSEARCH + Prosit-XL and xiSEARCH at an actual FDR of 1% at the CSM and peptide pair levels for the synthetic peptide dataset. **c)** Comparison of Scout+Prosit+XL+xiFDR and Scout (without xiFDR) on PPI level for synthetic protein dataset.

Supplementary Figure 4

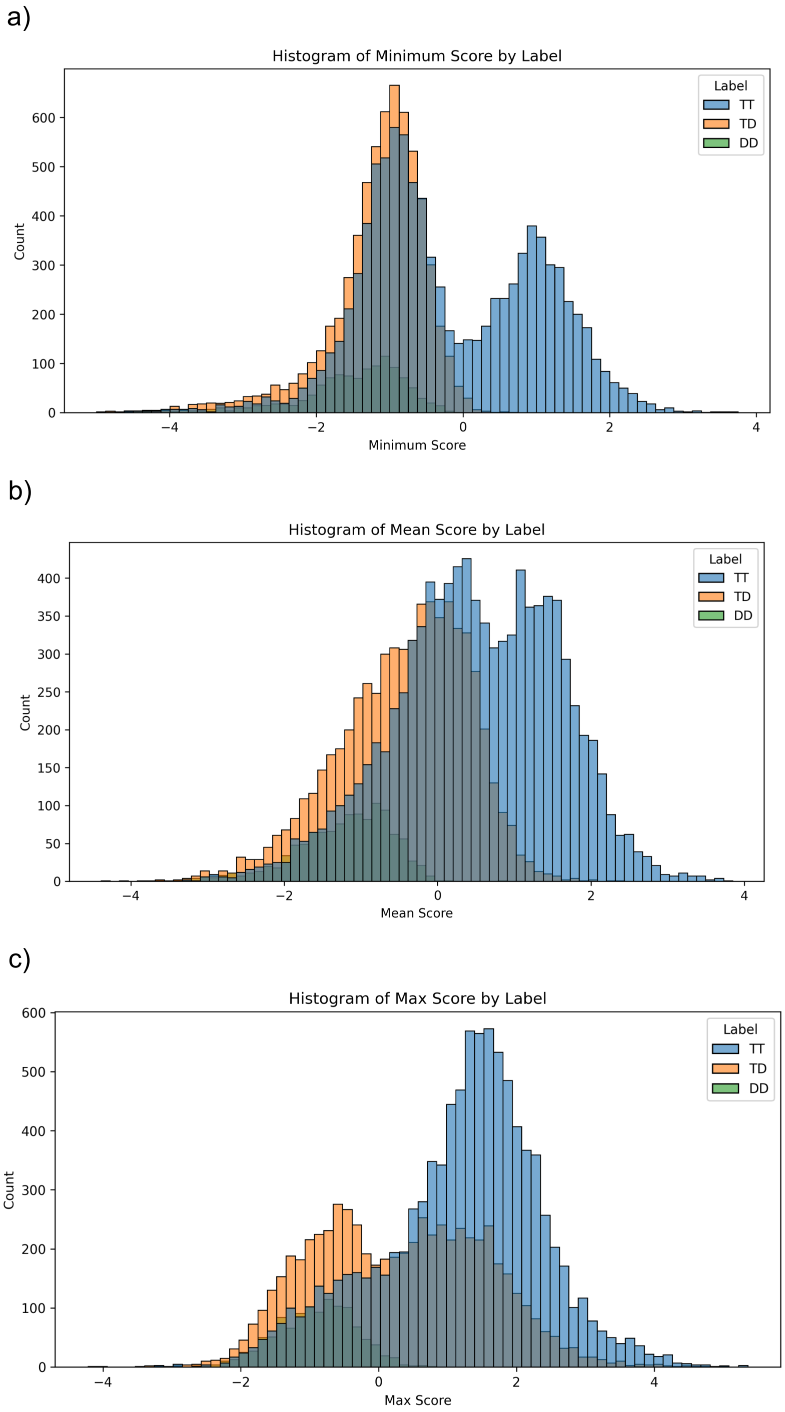

Supplementary Fig. 4 | Comparison of different methods for summarizing Percolator-optimized PSM-level scores into a single CSM-level score. Distributions of final CSM scores for target-target (TT), target-decoy (TD), and decoy-decoy (DD) matches in a synthetic peptide dataset (replicate 1) are shown using **a)** the minimum, **b)** the mean, and **c)** the maximum of the two associated PSM-level scores. Using the minimum score a) yields superior separation of true-positive TTs from TDs, DDs, and false-positive TTs, supporting its use as an effective scoring approach for downstream FDR estimation.

Supplementary Figure 5

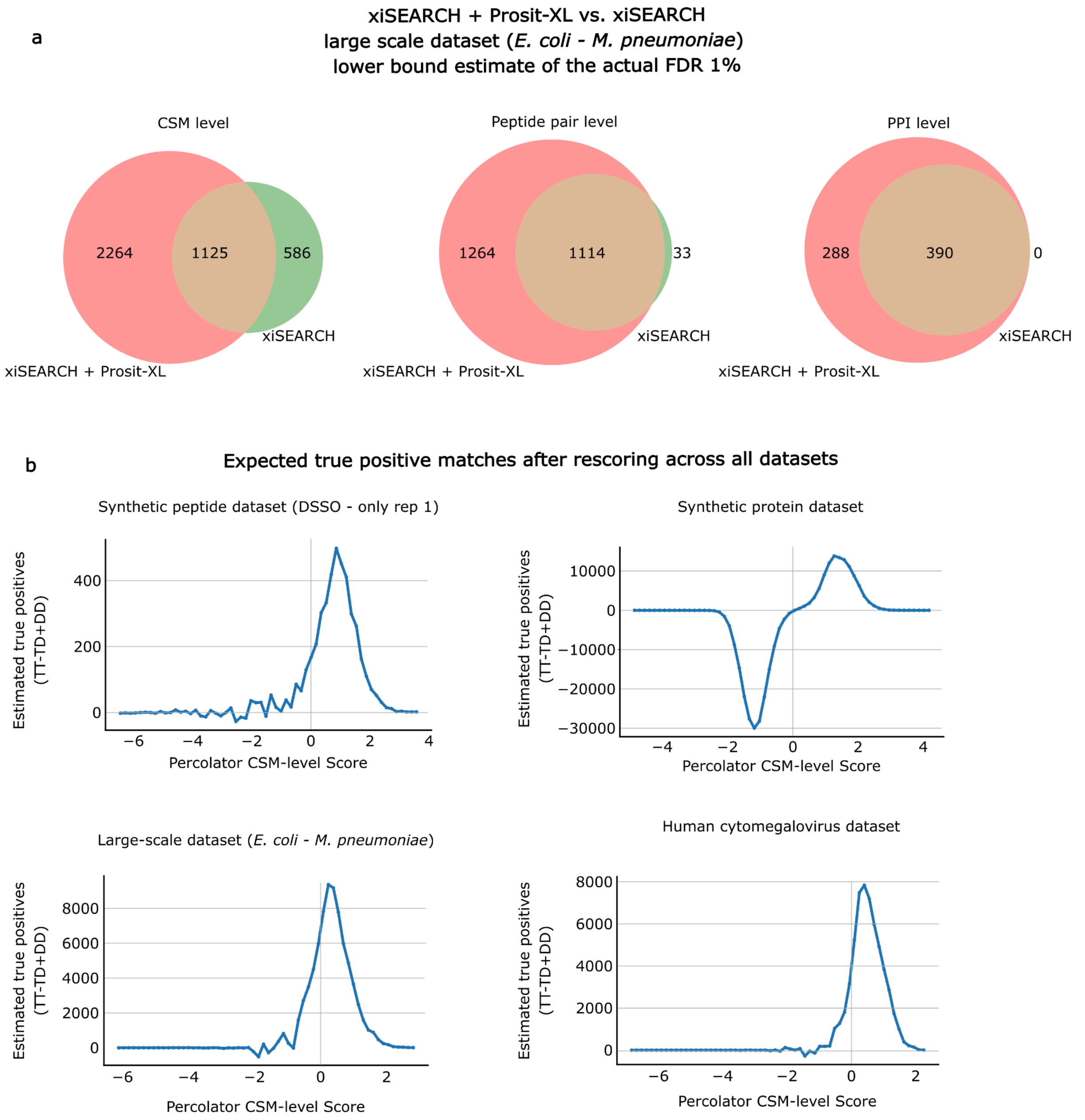

Supplementary Fig. 5 | a) Comparison of xiSEARCH + Prosit-XL and xiSEARCH at a lower bound estimate of the actual FDR of 1% at the CSM, peptide pair, and PPI levels for the combined large-scale dataset (E. coli – M. pneumoniae dataset). **b)** The number of expected true positive matches, calculated as #TT - (#TD - #DD), plotted against the Percolator CSM-level score across all analyzed datasets, including: synthetic peptide dataset, synthetic protein dataset, combined large-scale dataset (*E. coli - M. pneumoniae* dataset), and cytomegalovirus dataset.

Supplementary Figure 6

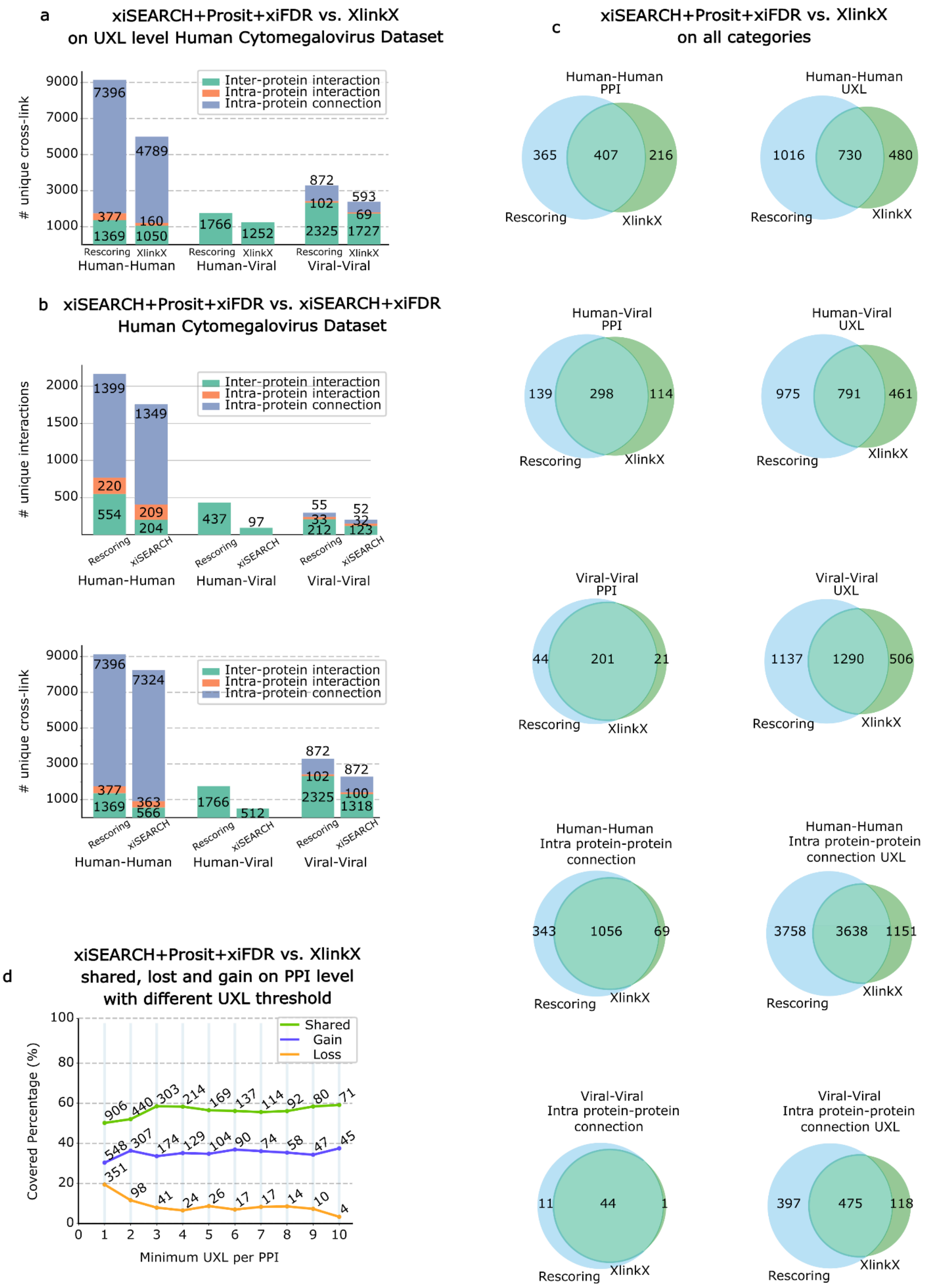

e Number of identified PPIs per each protein
(Inter Protein-Protein interaction)

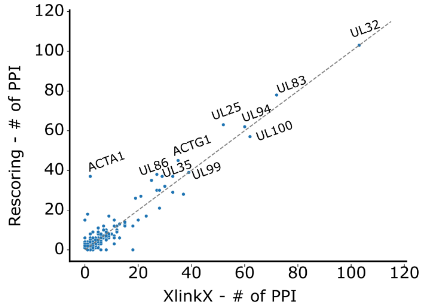

Top 10 UL83 PPIs by XlinkX	No. of UXLs by XlinkX	No. of UXLs by rescoring
UL32, UL83	62	96
UL25, UL83	44	62
UL83, UL83	37	45
UL45, UL83	37	38
UL83, UL99	34	44
**PPIA, UL83**	**33**	**36**
UL83, UL94	30	43
UL83, UL97	19	27
**DDX3X, UL83**	**15**	**34**
UL35, UL83	14	18

Top 10 UL25 PPIs by XlinkX	No. of UXLs by XlinkX	No. of UXLs by rescoring
UL25, UL83	44	62
UL25, UL45	18	22
UL25, UL25	18	23
UL25, UL32	17	35
UL25, UL99	16	22
UL25, UL35	16	18
**PPIA, UL25**	**11**	**17**
UL25, UL94	9	19
**GRB2, UL25**	**9**	**12**
**DDX3X, UL25**	**8**	**21**

f Distribution of distance and pLDDT scores
for human self-links

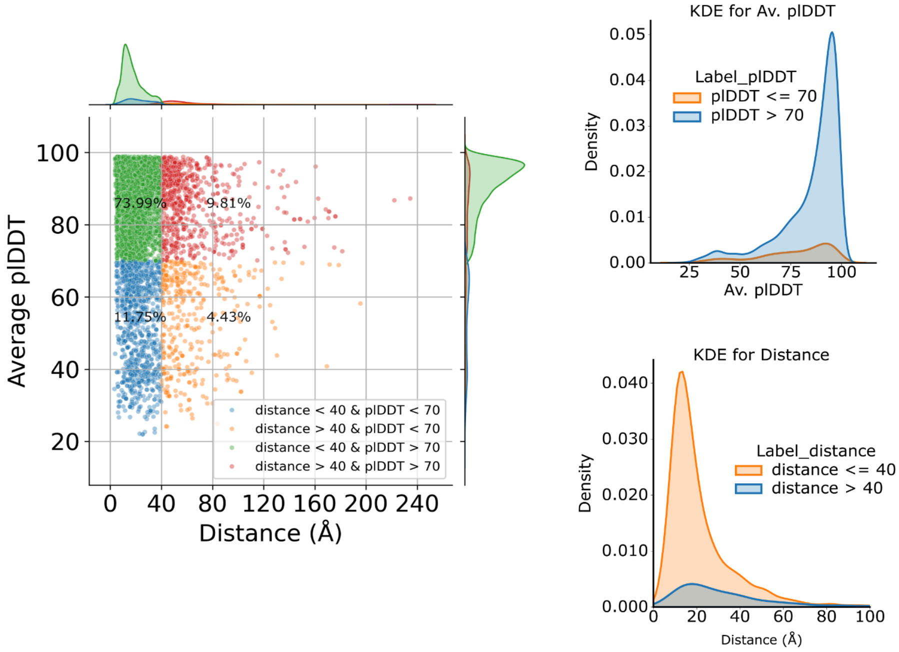

Supplementary Fig. 6 | Evaluation of data-driven rescoring on human cytomegalovirus virion dataset. a) The bars represent the number of UXLs for human-human, human-viral, and viral-viral interactions identified by xiSEARCH+Prosit-XL+xiFDR (left) and as reported in the original study by XlinkX (right). **b)** Unique number of identified interactions: inter protein-protein-interaction (green), intra protein-protein-interaction (orange), and intra protein-protein-connection (dark blue). The bars represent the number of PPIs and self-linkss for human-human, human-viral, and viral-viral interactions identified by data-driven rescoring of xiSEARCH+Prosit-XL+xiFDR results (left) and as xiSEARCH+xiFDR (right). **c)** Comparison of xiSEARCH+Prosit-XL+xiFDR and XlinkX on both PPI and UXL levels for all categories. **d)** Comparison of rescoring and XlinkX at the PPI level, showing shared, gained, and los t interactions when different thresholds of identified UXLs per PPI are applied. **e)** The plot represents the number of PPIs per protein (between-links) identified by XlinkX (x-axis) compared to those identified by rescoring (y-axis). The table below lists the top 10 PPIs identified by XlinkX for proteins UL83 and UL25, ranked by the number of UXLs, along with the corresponding UXL counts identified by rescoring. **f)** The left plot shows the UXL distances and plDDT scores of human self-links in four different groups including distance ≤ 40 Å and plDDT ≥ 70 (green points), distance ≤ 40 Å and Av. plDDT < 70 (blue points), distance > 40 Å and Av. plDDT ≥ 70 (red points), and distance > 40 Å and

Av. plDDT < 70 (orange points). The top right plot represents the kernel density estimate (KDE) distribution of plDDT scores for two groups: Av. plDDT < 70 (orange) and Av. plDDT > 70 (blue). The bottom right plot represents the KDE distribution of UXL distances for two groups: distance < 40 (orange) and distance > 40 (blue).

Supplementary Figure 7

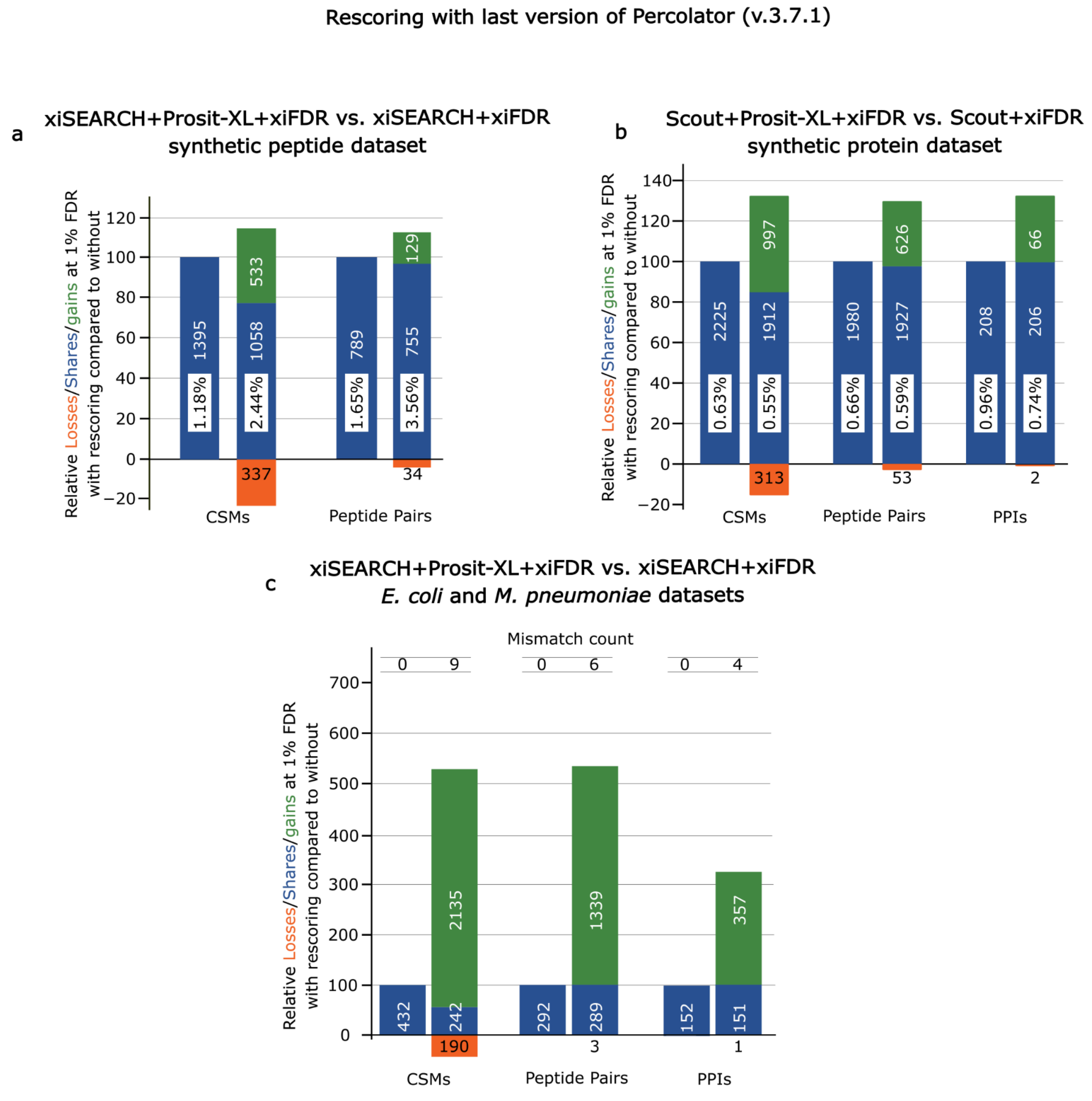

Supplementary Fig. 7 | Rescoring results using the last version of Percolator (v.3.7.1) on ground truth datasets. a) Vennbars show the number of identified CSMs and peptide pairs lost (orange), shared (blue), and gained (green), at an FDR of 1% on CSM- and peptide pair-levels when comparing results from xiSEARCH+Prosit-XL+xiFDR to xiSEARCH+xiFDR on a synthetic peptide dataset. Percentages inside the bars represent the actual FDRs, estimated by the ground truth synthetic peptide dataset. The analysis is based on both self- and between-link comparisons. **b)** Vennbars show the number of identified CSMs, peptide pairs, and PPIs lost (orange), shared (blue), and gained (green) at an FDR of 1% on CSM-, peptide pair-, and PPI-level when comparing results from Scout+Prosit-XL+xiFDR to Scout+xiFDR on a synthetic protein dataset. Percentages inside the bars represent the actual FDRs. The analysis is based on between-links only. **c)** Vennbars show the number of identified

CSMs, peptide pairs, and PPIs lost (orange), shared (blue), and gained (green) at an FDR of 1% on CSM-, peptide pair-, and PPI-level when comparing results from xiSEARCH+Prosit-XL+xiFDR (second bars) to xiSEARCH+xiFDR (first bars) on the results obtained from *E. coli - M. pneumoniae* dataset.

Supplementary Table 1

List of intensity-based features generated by Oktoberfest:

It is important to highlight that features are calculated separately for peptide A and B. In fact, no additional or cross-linking specific features are generated by Oktoberfest.

Number	Feature	Description
1	spectral_angle	Normalized spectral contrast angle (SA) on all potential y- and b-ions
2	pred_seen_nonzero	number of observed non-zero ions with non-zero predicted intensity
3	not_pred_seen	number of observed non-zero ions not predicted
4	not_pred_seen_b	number of observed non-zero b-ions not predicted
5	not_pred_seen_y	number of observed non-zero y-ions not predicted
6	pred_nonZero_fragments	number of ions with non-zero predicted intensity
7	pred_nonZero_b	number of b-ions with non-zero predicted intensity
8	pred_nonZero_y	number of y-ions with non-zero predicted intensity
9	pred_not_seen	number of ions with non-zero predicted intensity not observed
10	pred_not_seen_b	number of b-ions with non-zero predicted intensity not observed
11	pred_not_seen_y	number of y-ions with non-zero predicted intensity not observed
12	pred_seen_nonzero_b	number of observed b-ions with non-zero predicted intensity
13	pred_seen_nonzero_y	number of observed y-ions with non-zero predicted intensity
14	pred_seen_zero	number of predicted and observed zero-intensity ions
15	pred_seen_zero_b	number of predicted and observed zero-intensity b-ions
16	pred_seen_zero_y	number of predicted and observed zero-intensity y-ions

17	raw_nonZero_fragments	number of observed ions
18	raw_nonZero_b	number of observed b-ions
19	raw_nonZero_y	number of observed y-ions
20	rel_not_pred_seen	(number of observed ions not predicted) / (number of theoretically observable fragments [length*charge^2])
21	rel_not_pred_seen_b	(number of observed b-ions not predicted) / (number of theoretically observable fragments [length*charge])
22	rel_not_pred_seen_y	(number of observed y-ions not predicted) / (number of theoretically observable fragments [length*charge])
23	rel_pred_nonZero_b	(number of observed b-ions not predicted) / (number of theoretically observable fragments [length*charge])
24	rel_pred_nonZero_y	(number of observed y-ions not predicted) / (number of theoretically observable fragments [length*charge^2])
25	rel_pred_not_seen	(number of ions predicted, but not observed) / (number of theoretically observable fragments [length*charge^2])
26	rel_pred_not_seen_b	(number of b-ions ions predicted, but not observed) / (number of theoretically observable fragments [length*charge])
27	rel_pred_not_seen_y	(number of y-ions ions predicted, but not observed) / (number of theoretically observable fragments [length*charge])
28	rel_pred_seen_nonzero	(number of observed ions predicted) / (number of theoretically observable fragments [length*charge^2])
29	rel_pred_seen_nonzero_b	(number of observed b-ions predicted) / (number of theoretically observable fragments [length*charge])
30	rel_pred_seen_nonzero_y	(number of observed y-ions predicted) / (number of theoretically observable fragments [length*charge])

31	rel_pred_seen_zero	(number of predicted and observed zero-intensity ions) / (number of theoretically observable fragments [length*charge^2])
32	rel_pred_seen_zero_b	(number of predicted and observed zero-intensity b-ions) / (number of theoretically observable fragments [length*charge])
33	rel_pred_seen_zero_y	(number of predicted and observed zero-intensity y-ions) / (number of theoretically observable fragments [length*charge])
34	rel_raw_nonZero_fragments	(number of observed ions) / (number of theoretically observable fragments [length*charge^2])
35	rel_raw_nonZero_b	(number of observed b-ions) / (number of theoretically observable fragments [length*charge])
36	rel_raw_nonZero_y	(number of observed y-ions) / (number of theoretically observable fragments [length*charge])
37	relpred_not_pred_seen2pred_nonZero_fragments	(number of observed non-zero ions not predicted) / (number of ions with non-zero predicted intensity)
38	relpred_not_pred_seen_b2pred_nonZero_b	(number of observed non-zero b-ions not predicted) / (number of b-ions with non-zero predicted intensity)
39	relpred_not_pred_seen_y2pred_nonZero_y	(number of observed non-zero y-ions not predicted) / (number of y-ions with non-zero predicted intensity)
40	relpred_pred_nonZero_b2pred_nonZero_b	(number of b-ions with non-zero predicted intensity) / (number of b-ions with non-zero predicted intensity)
41	relpred_pred_nonZero_y2pred_nonZero_y	(number of y-ions with non-zero predicted intensity) / (number of y-ions with non-zero predicted intensity)
42	relpred_pred_not_seen_b2pred_nonZero_b	(number of b-ions with non-zero predicted intensity not observed) / (number of b-ions with non-zero predicted intensity)
43	relpred_pred_not_seen_y2pred_nonZero_y	(number of y-ions with non-zero predicted intensity not observed) / (number of y-ions with non-zero predicted intensity)

44	relpred_pred_not_seen2pred_nonZero_fragments	number of ions with non-zero predicted intensity not observed) / (number of ions with non-zero predicted intensity)
45	relpred_pred_seen_nonzero_b2pred_nonZero_b	(number of observed b-ions with non-zero predicted intensity) / (number of b-ions with non-zero predicted intensity)
46	relpred_pred_seen_nonzero_y2pred_nonZero_y	(number of observed y-ions with non-zero predicted intensity) / (number of y-ions with non-zero predicted intensity)
47	relpred_pred_seen_nonzero2pred_nonZero_fragments	(number of observed non-zero ions with non-zero predicted intensity) / (number of ions with non-zero predicted intensity)
48	relpred_pred_seen_zero_b2pred_nonZero_b	(number of predicted and observed zero-intensity b-ions) / (number of b-ions with non-zero predicted intensity)
49	relpred_pred_seen_zero_y2pred_nonZero_y	(number of predicted and observed zero-intensity y-ions) / (number of y-ions with non-zero predicted intensity)
50	relpred_pred_seen_zero2pred_nonZero_fragments	(number of predicted and observed zero-intensity ions) / (number of ions with non-zero predicted intensity)
51	pearson_corr	Pearson correlation on all potential y- and b-ions
52	spearman_corr	Spearman correlation on all potential y- and b-ions
53	mse	Mean square error
54	cos	Cosine similarity
55	std_abs_diff	Standard deviation of the absolute differences
56	abs_diff_Q3	Quantile 3 of the absolute differences
57	abs_diff_Q2	Quantile 2 of the absolute differences
58	abs_diff_Q1	Quantile 1 of the absolute differences
59	min_abs_diff	Minimum absolute difference
60	max_abs_diff	Maximum absolute difference

61	spectral_angle_single_charge	Normalized spectral contrast angle (SA) on singly charged ions
62	spectral_angle_double_charge	Normalized spectral contrast angle (SA) on doubly charged ions
63	spectral_angle_triple_charge	Normalized spectral contrast angle (SA) on triply charged ions
64	spectral_angle_b_ions	Normalized spectral contrast angle (SA) on b-ions
65	spectral_angle_y_ions	Normalized spectral contrast angle (SA) on y-ions
66	pearson_corr_single_charge	Pearson correlation on singly charged ions
67	pearson_corr_double_charge	Pearson correlation on doubly charged ions
68	pearson_corr_triple_charge	Pearson correlation on triply charged ions
69	pearson_corr_b_ions	Pearson correlation on b-ions
70	arson_corr_y_ions	Pearson correlation on y-ions
71	spearman_corr_single_charge	Spearman correlation on singly charged ions
72	spearman_corr_double_charge	Spearman correlation on doubly charged ions
73	spearman_corr_triple_charge	Spearman correlation on triply charged ions
75	spearman_corr_b_ions	Spearman correlation on b-ions
76	spearman_corr_y_ions	Spearman correlation on y-ions

Supplementary Note 1

Description of the process for running Percolator in Oktoberfest to rescore CSMs:

We propose a novel approach in which we use Percolator solely to generate an optimized score for each peptide precursor in a XL-peptide separately by running it on PSM level, rather than on CSM level. This is possible because Prosit-XL generates predictions for each peptide separately and circumvents modeling the four CSM score distributions for the possible true-positive (TP) and false-positive (FP) pairs (TP-TP, TP-FP, FP-TP, and FP-FP) that are present in the target-target (TT, covering TP-TP, TP-FP, FP-TP, and FP-FP), target-decoy (TD, covering TP-FP, FP-TP, and FP-FP) and decoy-decoy (DD, covering FP-FP) matches. When splitting up CSMs into two separate PSMs (one for peptide A and B, each), the clear notion of a target and decoy match remains. Further, the overall PSM-level score distribution of matches follows the expected behavior as known for linear peptides (Supplementary Fig. S3a). The result of this is a score that is optimized to separate correct from incorrect PSMs.

Because a CSM is incorrect when at least one of the two PSMs is incorrect, we pick the minimum Percolator-optimized PSM-level scores of the two PSMs associated with a CSM as a proxy for the quality of that CSM. This approach should be particularly effective for discriminating lower-scoring TP-TP pairs from higher-scoring TP-FP pairs, since the aggregate score (e.g. by summing) of TP-FP pairs might be overestimated by a good TP candidate. This is often the case in XL-MS, because one of the two peptides is dominating the fragmentation spectrum. In order to estimate CSM-, peptide pair-, and PPI-level FDR, the new CSM score is passed to xiFDR that can model the score distributions of TT, TD, and DD matches correctly.

To demonstrate the effectiveness of using the minimum Percolator-optimized PSM-level score to represent CSM quality, we compare it with other options, such as the mean and maximum scores (Supplementary Fig. S4). As an example, we show the distribution of the final CSM score on the x-axis versus TT, TD, and DD matches for the synthetic peptide dataset (rep1) using all three methods: minimum, mean, and maximum. The results clearly show that selecting the minimum score provides better differentiation between true-positive TTs and TDs, DDs, and false-positive TTs.

Supplementary Note 2

The list of UL55 cross-link distances for post-fusion (PDBID: 7KDD) and pre-fusion (PDBID: 7KDP):

Detected cross-links with distance >40 Å for Post-fusion, and <40 Å for Pre-fusion or with distance >40 Å for Pre-fusion, and <40 Å for Post-fusion. The data presented below outlines the distances between specific residues in both the pre-fusion and post-fusion configurations. For each residue, distances are given in the following format:
eg. "Structure, Chain1; Residue1, Chain2; Residue2, Distance"

Pre-fusion (>40 Å) - Post-fusion (<40 Å)

1. Post-fusion: 7kdd, Chain A; Residue 1: 568, Chain A; Residue2: 670, Distance Å: 107.4
1. Pre-fusion: 7kdp, Chain A; Residue 1: 568, Chain A; Residue2: 670, Distance Å: 28.7
2. Post-fusion: 7kdd, Chain A; Residue 1: 535, Chain B; Residue2: 670, Distance Å: 77.6
2. Pre-fusion: 7kdp, Chain A; Residue 1: 535, Chain B; Residue2: 670, Distance Å: 32.4
3. Post-fusion: 7kdd, Chain A; Residue 1: 378, Chain B; Residue2: 88, Distance Å: 69.1
3. Pre-fusion: 7kdp, Chain A; Residue 1: 378, Chain B; Residue2: 88, Distance Å: 27.7
4. Post-fusion: 7kdd, Chain A; Residue 1: 670, Chain C; Residue2: 88, Distance Å: 85.4
4. Pre-fusion: 7kdp, Chain A; Residue 1: 670, Chain C; Residue2: 88, Distance Å: 32.1
5. Post-fusion: 7kdd, Chain A; Residue 1: 568, Chain A; Residue2: 691, Distance Å: 135.3
5. Pre-fusion: 7kdp, Chain A; Residue 1: 568, Chain A; Residue2: 691, Distance Å: 32.2
6. Post-fusion: 7kdd, Chain A; Residue 1: 535, Chain C; Residue2: 691, Distance Å: 100.4
6. Pre-fusion: 7kdp, Chain A; Residue 1: 535, Chain C; Residue2: 691, Distance Å: 30.6
7. Post-fusion: 7kdd, Chain A; Residue 1: 568, Chain A; Residue2: 695, Distance Å: 142.6
7. Pre-fusion: 7kdp, Chain A; Residue 1: 568, Chain A; Residue2: 695, Distance Å: 31.4
8. Post-fusion: 7kdd, Chain A; Residue 1: 535, Chain C; Residue2: 695, Distance Å: 108.3
8. Pre-fusion: 7kdp, Chain A; Residue 1: 535, Chain C; Residue2: 695, Distance Å: 36.1
9. Post-fusion: 7kdd, Chain A; Residue 1: 535, Chain A; Residue2: 695, Distance Å: 148.2
9. Pre-fusion: 7kdp, Chain A; Residue 1: 535, Chain A; Residue2: 695, Distance Å: 41.6

Pre-fusion (<40 Å) - Post-fusion (>40 Å)

1. Post-fusion: 7kdd, Chain A; Residue 1: 535, Chain C; Residue2: 695, Distance Å: 21.8
1. Pre-fusion: 7kdp, Chain A; Residue 1: 535, Chain C; Residue2: 695, Distance Å: 52.8
2. Post-fusion: 7kdd, Chain A; Residue 1: 209, Chain C; Residue2: 700, Distance Å: No info
2. Pre-fusion: 7kdp, Chain A; Residue 1: 209, Chain C; Residue2: 700, Distance Å: 44.6
3. Post-fusion: 7kdd, Chain A; Residue 1: 378, Chain C; Residue2: 670, Distance Å: 26.0
3. Pre-fusion: 7kdp, Chain A; Residue 1: 378, Chain C; Residue2: 670, Distance Å: 58.1
