# Supplementary material for: Prosit-XL: enhanced cross-linked peptide identification by fragment intensity prediction to study protein interactions and structures
Source: Nat Commun. 2025 Jul 1;16:5429. doi: 10.1038/s41467-025-61203-4 (PMC12214610; doi:10.1038/s41467-025-61203-4)
Supplement: Supplementary file 2 — Description of Additional Supplementary Files [file 41467_2025_61203_MOESM2_ESM.pdf]

Description of Additional Supplementary Files

File name: Supplementary Data 1

Description: The name of MS files used for data collection (training, validation, and holdout set) as well as those used in the rescoring process for each dataset.

File name: Supplementary Data 2

Description: The output of xiFDR (identified CSMs, peptide pairs, and PPIs) for the synthetic peptide dataset.

File name: Supplementary Data 3

Description: The output of xiFDR (identified CSMs, peptide pairs, and PPIs) for the synthetic protein dataset.

File name: Supplementary Data 4

Description: The output of xiFDR (identified CSMs, peptide pairs, and PPIs) for the E. coli and M. pneumoniae dataset.

File name: Supplementary Data 5

Description: The output of xiFDR (identified CSMs, peptide pairs, and PPIs) for the human cytomegalovirus dataset.
